# Are the poorest poor being left behind? Estimating global inequalities in reproductive, maternal, newborn and child health

**DOI:** 10.1136/bmjgh-2019-002229

**Published:** 2020-01-26

**Authors:** Aluisio J D Barros, Fernando C Wehrmeister, Leonardo Zanini Ferreira, Luis Paulo Vidaletti, Ahmad Reza Hosseinpoor, Cesar G Victora

**Affiliations:** 1 Department of Data and Analytics, WHO, Geneve, Switzerland; 2 International Center for Equity in Health, Universidade Federal de Pelotas, Pelotas, Brazil; 3 Post-Graduate Programme in Epidemiology, Universidade Federal de Pelotas, Pelotas, Brazil

**Keywords:** child health, maternal health

## Abstract

**Introduction:**

Wealth-related inequalities in reproductive, maternal, neonatal and child health have been widely studied by dividing the population into quintiles. We present a comprehensive analysis of wealth inequalities for the composite coverage index (CCI) using national health surveys carried out since 2010, using wealth deciles and absolute income estimates as stratification variables, and show how these new approaches expand on traditional equity analyses.

**Methods:**

83 low-income and middle-income countries were studied. The CCI is a combined measure of coverage with eight key reproductive, maternal, newborn and child health interventions. It was disaggregated by wealth deciles for visual inspection of inequalities, and the slope index of inequality (SII) was estimated. The correlation between coverage in the extreme deciles and SII was assessed. Finally, we used multilevel models to examine how the CCI varies according to the estimated absolute income for each wealth quintile in the surveys.

**Results:**

The analyses of coverage by wealth deciles and by absolute income show that inequality is mostly driven by coverage among the poor, which is much more variable than coverage among the rich across countries. Regardless of national coverage, in 61 of the countries, the wealthiest decile achieved 70% or higher CCI coverage. Well-performing countries were particularly effective in achieving high coverage among the poor. In contrast, underperforming countries failed to reach the poorest, despite reaching the better-off.

**Conclusion:**

There are huge inequalities between the richest and the poorest women and children in most countries. These inequalities are strongly driven by low coverage among the poorest given the wealthiest groups achieve high coverage irrespective of where they live, overcoming any barriers that are an impediment to others. Countries that ‘punched above their weight’ in coverage, given their level of absolute wealth, were those that best managed to reach their poorest women and children.

**Key questions**

**What is already known?**

- Inequalities in health have been documented extensively, especially using survey-based analysis using wealth quintiles.
- Socioeconomic inequalities in health intervention coverage have been reduced in several settings but remain wide in many countries.

**What are the new findings?**

- We found marked socioeconomic inequalities in the composite coverage index (CCI) within most countries, which are more obvious when comparing wealth deciles instead of the traditionally used wealth quintiles.
- The level of inequality in countries is strongly correlated to coverage among the poorest 10% of the population. We found much more marked variability in coverage levels between countries among the poorest than among the richest. Monitoring coverage in the poorest decile in each country is a simple way of auditing progress in both coverage and equity.
- Countries vary widely in terms of coverage achieved for similar levels of wealth in their populations. Countries that achieve high coverage among the poorest women and children are also those with the lowest levels of overall inequality.

**What do the new findings imply?**

- Our findings indicate that assessing country progress towards the health SDGs will be better served by looking at coverage among the poor than reporting on national coverage.
- Regular analyses of coverage by wealth deciles and by absolute levels of wealth will provide essential inputs to monitoring, benchmarking and health programming.
- For national level programming and policy making, our results make clear that future success will largely depend on whether the poorest women and children are being effectively reached with essential interventions.

## Introduction

The Sustainable Development Goals (SDG) motto—‘Leave no one behind’—is a clear call for progress with equity. For this purpose, SDG 17.18 specifically requires disaggregated analyses of available indicators to ensure that all population subgroups are benefitting from national level progress. In terms of the health indicators that fall under SDG 3, disaggregated analyses focused on wealth have typically relied on stratifying households in five groups, from the 20% poorest to the 20% richest households. Commonly, in national health surveys, data on household assets and building materials are used to estimate an asset index score that is used to rank households and classify them into five equally sized groups—the wealth quintiles.^1 2^ Such analyses have systematically revealed important gaps between rich and poor in most countries and allowed monitoring of inequalities over time and targeting of interventions.

However, dividing the households into five equally sized groups may be convenient in terms of interpretation, and producing adequate sample sizes, but may mask important differences in health intervention coverage within each group.^3^ These differences may be largest in the extreme groups, the poorest or the richest, depending on the setting. A more granular approach may help reveal and understand wealth-related inequalities beyond what can be detected by quintiles. Dividing households into 10 wealth groups, or deciles, is a viable alternative to reveal further information on inequalities, still keeping sample sizes sufficiently large for robust estimation.^3^ Using this approach, it is common to find that the poorest 10% of women and children tend to lag well behind the second poorest decile and the rest of the population or that those in the richest decile are way ahead of the rest.

Yet, a recurring critique of asset indices is that these represent a relative measure of wealth, which are well suited for comparisons within a country but not between countries. For example, the poorest quintile in a middle-income country may be wealthier than, say, the third quintile in a low-income country. To address this issue, a strategy was developed to attach absolute dollar values to wealth quintiles (or any other quantiles, such as deciles or centiles) in a given country at a given point in time.^4^ This brings an innovative perspective to between-country comparisons, enabling the comparison of countries in terms of health outcomes allowing for absolute wealth levels of population subgroups.

With these tools in hand, we report on wealth-related inequalities in the composite coverage index (CCI). This is a summary measure of coverage with health interventions for reproductive, maternal, newborn and child health (RMNCH), which represents a proxy for universal health coverage for women and children and which has been widely used in monitoring country progress towards the Millennium Development Goals or MDGs (2000–2015) and the SDGs (2015–2030).^5–7^ We analysed national surveys carried out since 2010, from 83 low-income and middle-income countries (LMICs) for which suitable data were available. We report on inequalities by wealth deciles by looking at gaps in coverage between the 10% poorest and 10% richest within each country and by analysing how much coverage varies among the poorest and richest groups across countries. Finally, we explored how levels of coverage vary at comparable levels of income in different countries to identify which countries are reaching high coverage for their poorest women and children, even in the presence of widespread destitution.

## Methods

Household surveys are the main source of information on RMNCH for LMICs; Demographic Health Surveys (DHS; www.dhsprogram.com) and Multiple Indicator Cluster Surveys (MICS; mics.Unicef.org) are the main global programmes running such surveys. These surveys employ multistage sampling strategies, with clusters (usually census tracts) being the primary sampling units. We analysed all standard DHS and MICS surveys carried out from 2010 that were representative at national level and had suitable data to calculate the wealth index and the CCI. For countries with multiple surveys, we used the most recent one for which the data were publicly available.

In order to monitor progress on the MDGs and SDGs, the Countdown to 2030 initiative (www.countdown2030.org) has developed the CCI.^5 6^ It was initially proposed in 2008 as the ‘coverage gap’. In the RMNCH literature, the CCI has been increasingly used as a proxy indicator of universal health coverage, being strongly and inversely correlated with under-five mortality and other relevant outcomes.^7^ It has been used often in publications exploring health inequalities and monitoring progress with universal health coverage.^8–11^

The CCI was our main outcome. It is a weighted average of coverage levels with eight health interventions along four stages of the RMNCH continuum of care (reproductive health; maternal and newborn health; preventive child interventions; and case management of childhood illnesses), with weights giving equal importance to each stage. The expression used for the calculation is

$$CCI=\frac{1}{4}\left(DFPSm+\frac{ANC4+SBA}{2}+\frac{BCG+2DPT3+MCV}{4}+\frac{ORS+CPNM}{2}\right)$$

where DFPSm is demand for family planning satisfied with modern methods, ANC4 is 4+ antenatal care visits, SBA is skilled birth attendant, BCG is one dose of BCG vaccine, DPT3 is 3+ doses of DPT vaccine (or a polyvalent vaccine containing DPT), MCV is one or more doses of a measles-containing-vaccine, ORS is oral rehydration salts for diarrhoea and CPNM is care-seeking for suspected pneumonia. The full definition of each indicator is presented in the online supplementary table S1, supplemental annex 1, along with more details on the methods used in this manuscript. The CCI SEs were calculated through bootstrapping.

10.1136/bmjgh-2019-002229.supp1 Supplementary data

The wealth classification used in the analyses is based on an asset index created through principal components analysis,^12^ accounting for differences in the importance of assets between urban and rural households. The variables used to calculate the score include household assets (eg, cookstove, bicycle and car), building materials of the house (eg, wood floor, brick walls and corrugated roof) and access to utilities (eg, sanitation and electricity). The score is provided with the original survey datasets and calculated according to a standard methodology.^13 14^ The households in the surveys are ranked according to the resulting score and split into five or ten equally sized groups (quintiles or deciles).

The attribution of absolute income values to households was done according to the methods described in Fink *et al*.^4^ In summary, the income distribution for each country is estimated by using the consumption share of the country’s gross domestic product (GDP) and the Gini coefficient to generate the parameters for a log-normal distribution.^15 16^ This allows the estimation of average absolute income in dollars for each quintile of the population. Next, the households in the survey sample are ranked by the asset index, and all households in each wealth quintile (or decile) are assigned the dollar value corresponding to the same quintile of the income distribution. Income is expressed in constant 2011 international dollars adjusted at purchasing power parity.

Inequality across the wealth distribution was measured using the slope index of inequality (SII), based on the values of the CCI stratified by wealth quintiles. A logistic regression model is fitted with the CCI for each quintile as the dependent variable and mean group fractional rank as the predictor. Since the quintiles are equally sized, the ranks are equal to 0.1, 0.3, 0.5, 0.7 and 0.9 for the five quintiles ordered from poorest to richest. The difference between the extremes of the wealth distribution is then estimated using the resulting model by estimating the difference between the fitted values for rank 1 and zero. When used for coverage indicators, the SII can vary from −100 to +100, zero meaning absence of inequality. A positive value indicates that the richer groups present higher coverage than the poorer groups, while a negative value means the opposite. The SII estimated value represents the difference between theoretical top and bottom of the wealth distribution and represents a measure of absolute rather than relative inequality.^6^ Earlier analyses showed that the values of the SII calculated by wealth quintiles are essentially equal to those calculated by wealth deciles.^3^

The estimation of the expected (average) CCI coverage for a given level of absolute income in international dollars, by combining all 83 surveys, was done through a linear multilevel model where the outcome was the CCI and the predictor was the log-transformed income. Quintiles were level 1 units and countries were level 2 units in the multilevel model.

Due to the stratification process by wealth quintiles or deciles, in some groups, we do not have data to estimate the CCI. Most often this is due to no observed children with pneumonia, so that the indicator for careseeking for suspected pneumonia cannot be estimated. In the few cases it happened in this analysis (7 out of 830 deciles), these data points were excluded from the analysis.

All analyses were carried out with Stata V.15. The individual level analyses (such as estimation of the CCI and components) took into account the survey design. This includes adjusting for the clustered sample, stratification and using sample weights. Country-level (or quintile-level) analyses were not weighted by population size so that each country (or quintile) has the same weight in the analyses (eg, correlation estimates).

### Patient and public involvement

Patients were not involved in the design, or conduct, or reporting, or dissemination plans of our research. The study is based on publicly available data based on national health surveys.

## Results

Using data from available surveys, we were able to estimate all eight indicators required for the CCI for 83 countries from all seven UNICEF world regions. This included 21 countries from Western and Central Africa, 17 countries from Eastern and Southern Africa, 8 countries from the Middle East and North Africa, 10 countries from Europe and Central Asia, 6 countries from South Asia, 9 countries from East Asia and the Pacific and 12 countries from Latin America and the Caribbean. The oldest survey was from 2010, and the most recent was from 2017, with the median year being 2014. The country list grouped by UNICEF world regions is presented in online supplementary table S1, in the supplemental annex 2.

**Figure 1:**
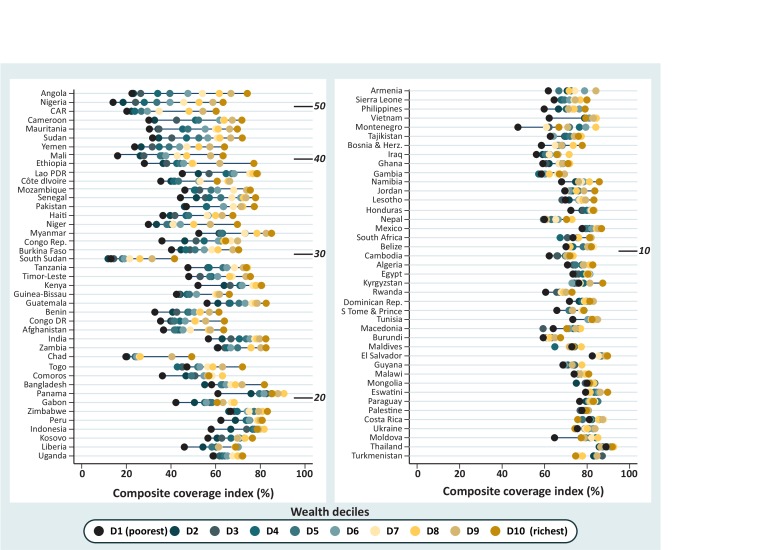
Equiplot showing the coverage with the composite coverage index by wealth deciles for the 83 study countries ordered by decreasing inequality. The depth marks indicate the range of inequality in percentage points measured by the slope index of inequality. DHS and MICS, 2010–2017. DHS, Demographic Health Surveys; MICS, Multiple Indicator Cluster Surveys.

Figure 1 shows the CCI values by wealth deciles in these 83 countries. Countries are ordered from the highest to lowest level of inequality, measured by the SII. The depth marks show the levels of SII: 19 countries present gaps wider than 30 percentage points (p.p.). In two countries, Angola and Nigeria, this gap is greater than 50 p.p. The 19 countries with an SII greater than 30 p.p. include 10 from Western and Central Africa. The CCI estimates at national level and by deciles and the SII for each country are presented in online supplementary table S2, in the supplemental annex 2.

Looking at the distribution of coverage for each decile, it is possible to identify several cases of what is described as ‘bottom inequality’, where the poorest groups are way behind the rest.^17^ Mali, Ethiopia, Myanmar, the Republic of Congo, Tanzania, Kenya, Benin, Comoros, Panama, Gabon, Peru, Indonesia, Liberia, Vietnam, Montenegro and Moldova are the countries where this pattern is more evident (note that Montenegro does not have an estimate for the second decile due to lack of data, and this may exaggerate the bottom inequality pattern). In all of these cases, the 10% poorest fare much worse than the second or wealthier deciles. Moldova is an interesting case of a country with high coverage and low inequality, and still the poorest 10% of the population stand out with much lower coverage than the rest. Yet, even in countries with huge inequality, it is often impossible to pinpoint a single group that is left way behind as deciles are spread more or less evenly over the coverage range.

**Figure 2:**
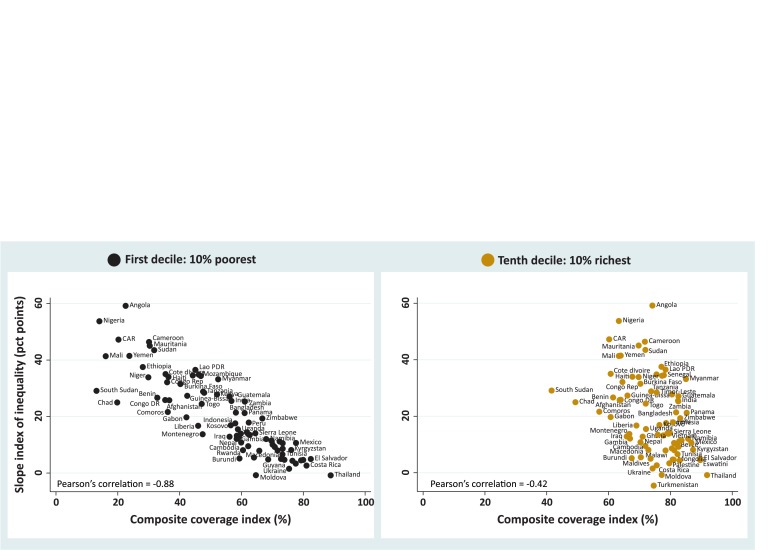
Slope index of inequality versus CCI coverage for the poorest 10% and the richest 10% households in each of the 83 study countries with the respective regression lines plotted. DHS and MICS, 2010–2017. CCI, composite coverage index; DHS, Demographic Health Surveys; MICS, Multiple Indicator Cluster Surveys.

In figure 2, we further explore a feature that can already be visually identified in figure 1. Between-country variability in coverage among the poorest is much more marked than variability among the richest. The left-hand-side graph in figure 2 shows a very strong inverse correlation (−0.88, p<0.001) between CCI in the poorest decile and the national inequality level expressed by the SII. The right-hand graph shows that the corresponding correlation for the richest decile is much weaker (−0.42, p<0.001). That is, the lower the coverage among the poorest, the higher the national level of inequality. This is less true for the richest, for whom the median CCI is 75% and between-country variability is much smaller than for the poorest (median coverage 59%). (See the box and whisker plot presented in online supplementary figure S1, supplemental annex 2, for more details). There are only three exceptions where coverage for the richest 10% is below 60%—Comoros, Chad and South Sudan, all three from sub-Saharan Africa.

**Figure 3:**
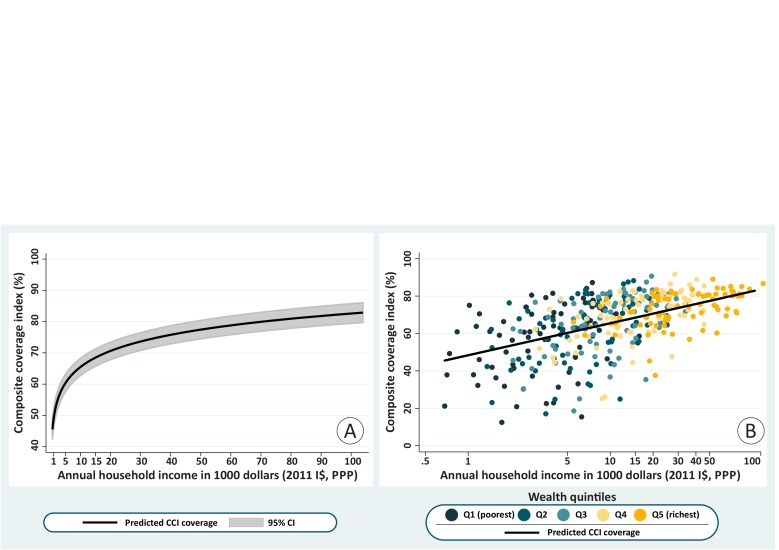
Composite coverage index (CCI) versus annual household income (2011 international dollars at purchasing power parity (PPP)) fitted through multilevel linear model. Figure 3A (left) presents the predicted line on a linear scale with its 95% CI. Figure 3B (right) presents the predicted line plus all wealth quintiles in each country on a log scale. DHS and MICs, 2010–2017. DHS, Demographic Health Surveys; I$, international dollars; MICS, Multiple Indicator Cluster Surveys.

We then used the values of absolute income attributed to each wealth quintile to predict expected CCI coverage levels. Given the lack of information on either GDP or the Gini index, we could not estimate absolute income for three countries (Maldives, Sao Tome & Principe and Yemen). Using 400 quintiles from 80 countries (listed in online supplementary table S1, supplemental annex 2), we estimated the expected coverage for a given household income level. The prediction line for CCI by income resulting from the multilevel model is presented in figure 3A. There is a steep increase in CCI from the lowest incomes (less than $1000 per year) up to around $15 000. The lowest predicted coverage is 45% for an income of $680, and it increases to 68% for an income of $15 000. Above this income, the increase in CCI is less steep, reaching 83% at an income of approximately $100 000.

Figure 3B shows the prediction line in log scale (given that the income variable is strongly skewed), plus the observed data points (quintiles). The CCI increases linearly with log income, and the model implies that, over all countries, every time income doubles, CCI coverage increases by 5.2 p.p. (95% CI 4.7 to 5.6). It is also clear from the figure that the variability in CCI decreases with income, in accordance with what we described in the analysis by wealth deciles.

**Figure 4:**
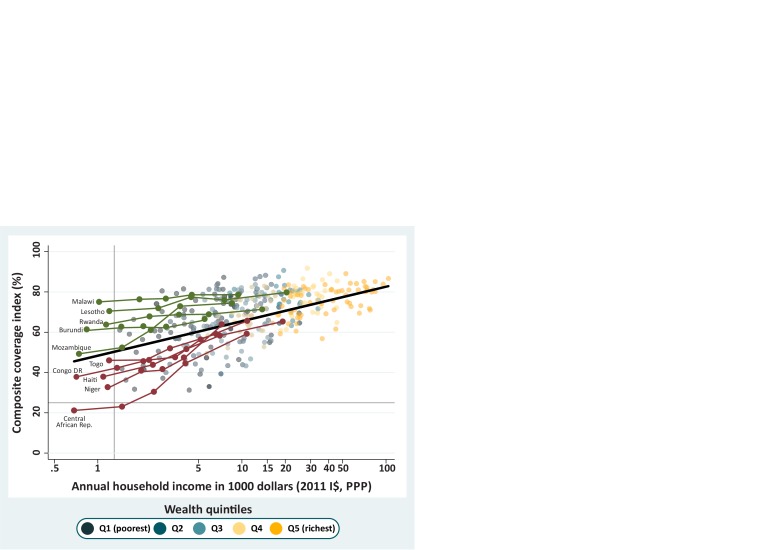
Countries with an income less than $1300 in the poorest quintile are highlighted in green when they present a higher-than-expected composite coverage index (CCI) and in Burgundy when they present a CCI coverage lower than expected. The black line represents the expected CCI coverage for a given level of income, and all quintiles are presented as shaded dots in the background. DHS and MICs, 2010–2017. DHS, Demographic Health Surveys; I$, international dollars; MICS, Multiple Indicator Cluster Surveys; PPP, purchasing power parity.

We highlighted two groups of countries in figure 4 using a criterion of poverty. We chose countries where income was below 1300 dollars in the poorest quintile (Q1). This cut-off selects just over 10% of the countries with data and was chosen because this value marks a split in the income distribution of Q1, where there is a gap separating the poorest first quintiles from the rest. The highlighted countries are shown in figure 4 in two different colours. In green, we highlighted the countries with very low income in Q1 where the poorest quintiles presented higher coverage than expected according to the regression line. The two countries with the highest coverage for Q1 were Malawi and Lesotho, both from Eastern and Southern Africa. In contrast, countries in red are those where the poorest quintiles present lower coverage than expected. Central African Republic (CAR) and Niger from Western and Central Africa show the lowest CCI coverages for Q1 in this group. In figure 4, coverage inequality affects the slope of the country lines or the vertical spread of the quintiles. Countries in green, with higher coverage than expected among the poor, are much more equitable than countries in red. In all cases, coverage is higher among the richest women and children, and values for all countries tend to be closer to the expected coverage level than for the poor.

**Figure 5:**
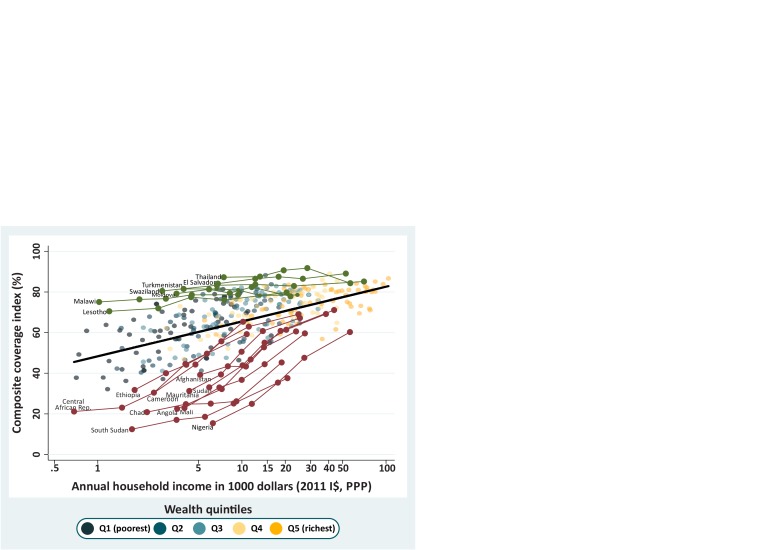
Countries in green are those with a composite coverage index (CCI) more than 20 percentage points higher than expected for the poorest quintile and those in Burgundy had a CCI coverage less than 20 percentage points than expected. The black line represents the expected CCI coverage for a given level of income, and all quintiles are presented as shaded dots in the background. DHS and MICs, 2010–2017. DHS, Demographic Health Surveys; I$, international dollars; MICS, Multiple Indicator Cluster Surveys; PPP, purchasing power parity.

In our last step of the analyses, we singled out all countries where coverage for the poorest quintile was more than 20 p.p. above or below the expected value (figure 5), and identified 11 underachievers and seven overachievers, respectively. The top overachiever was Malawi, with CCI 27 p.p. above the expected value for the poorest quintile, followed by Eswatini, Thailand, Turkmenistan, El Salvador, Moldova and Lesotho.

With lower coverage than expected, we had Nigeria 47 p.p. below the expected CCI, followed by South Sudan, Mali, Angola, Chad, Sudan, Mauritania, Cameroon, CAR, Afghanistan and Ethiopia. Among this set of countries are some with the highest inequality levels, such as Angola and Nigeria, which were the countries with the highest SII (59.2 and 53.7 p.p., respectively). Mali, South Sudan and Chad also present high SII values of 41.4, 29.1 and 25.0, respectively. All the differences between the observed and expected values are presented in online supplementary table S3, supplemental annex 2.

Figure 5 clearly shows that the overachievers include countries where inequality is narrow (as shown by the smaller vertical variability by quintile) and where all quintiles show a CCI value above of what was expected on the basis of income. In agreement with the earlier findings by deciles, figure 5 suggests that greater equity is achieved by higher-than-expected coverage among poorer women and children.

The analysis using absolute income reinforces the findings that levels of coverage among the poorest vary hugely and, more importantly, that some countries manage to achieve markedly higher coverage than others, for similar levels of income. In a comparison of extremes, Malawi had a CCI coverage of 73.9% for the poorest quintile with an annual household income of $759, while South Sudan had a CCI of 11.8% in the poorest quintile while the annual income was $2213.

## Discussion

In the present set of global analyses, we explored new approaches to describing and understanding wealth-related inequalities in intervention coverage using a combined indicator—the CCI. Our results confirm the value of the CCI for global comparisons; as a single summary measure, it is easy to calculate, present and interpret. Its advantages have been highlighted in previous publications regarding its theoretical basis, robustness and validity.^6 7^

Our exploration of inequalities in 83 LMICs revealed 19 countries with very large inequalities, where we observed an SII of 30 or more percentage points. Out of these 19 countries, 13 were from sub-Saharan Africa. Angola and Nigeria stood out with SII values greater than 50 p.p. Another country that calls for attention to wide disparities was Haiti, particularly because most of the countries in the same region—Latin America and the Caribbean—present lower levels of inequality.

The examination of deciles also revealed some countries—such as Congo, Ethiopia, Mali and Myanmar—where inequality was wide and where the poorest decile has coverage levels well below the second decile. It is likely that future studies examining even more extreme groups in terms of poverty—such as the poorest 5% or 1%—will reveal even lower coverage levels in most settings. There is a limit to such analyses, however, which is imposed by survey sample sizes and poor precision for small group estimates.^3^ Decile analyses represent a reasonable compromise for identifying settings where the poorest are way behind the other groups, with sufficiently precise estimates.

Our analyses of RMNCH coverage by wealth decile and by absolute income provide compelling evidence showing that inequality is primarily driven by coverage among the poorest women and children. Regardless of the overall national coverage, in 61 of all countries studied, the wealthiest decile of women and children have reached 70% or higher CCI coverage. However, we observed wide variability in coverage for the poorest deciles. It is mostly low coverage for the poorest that characterises the countries with the largest gaps, given the richest have higher and less variable levels of coverage. This suggests that, independent of where they live, the better-off can overcome the most common barriers to access services through their purchasing power, better support networks or better social connections.

These barriers will vary from place to place, being directly related to the ability of the richer women and children to have geographical, cultural and economic access to health services and to have access to information on the value of interventions and on how services work.^18^

These findings also indicate that assessing country progress towards the health SDGs would be better served by looking at coverage among the poor than relying on national coverage levels, as has been previously argued in the MDG era.^19^ It is remarkable that none of the health SDG targets relate to specific reductions in inequality, in spite of the lip service to leaving no one behind. For national level programming and policy making, our results make clear that future success will largely depend on whether the poorest women and children are being effectively reached with essential interventions. The Pan-American Health Organization provides an example to other international agencies by stating its regional goals in terms of reductions in national levels of maternal and infant mortality and in reducing the absolute and relative gaps.^20^

Our analyses of coverage according to absolute wealth overcame many of the limitations of using relative quintiles when comparing countries^21^ and have allowed benchmarking of progress at national level and for socioeconomic subgroups of the population. With these analyses, we were able to produce results that have not been previously described in the literature. Well-performing countries, those which ‘punched above their weight’ in coverage allowing for wealth, did so by being particularly effective in achieving high coverage among the poor. In contrast, underperforming countries failed to reach the poorest women and children, in spite of reaching the better-off, for the most part.

It is also important to note that highly unequal countries, with some of the lowest coverage levels for the poorest, are not the poorest countries. Angola, Chad, Mali, Nigeria and South Sudan tended to present much lower than expected coverage levels for all wealth groups, even for the richest. These findings warrant in-depth investigations on the drivers of coverage and equity in these contexts. It is noteworthy that underachievers such as South Sudan and Nigeria have been affected by prolonged humanitarian crises, adding to other barriers linked with political instability and economic crises. An earlier national-level analysis showed that conflict and violence were major drivers of CCI inequalities.^22^ Good governance and health system organisation and effectiveness, however, constitute positive drivers for improvement.

Although the CCI provides an overall view of RMNCH intervention coverage, it fails to reveal what are the main bottlenecks in each country. The present analyses may be expanded on to study specific components of the CCI that are delivered by different channels, for example, skilled birth attendance,^3 23^ which largely relies on access to health facilities, in comparison with immunisations that are often delivered at community level. Such detailed analyses are beyond the scope of the present paper.

Our study has other limitations. The use of wealth deciles may be affected by small sample sizes for some surveys. However, the number of deciles for which we could not estimate the CCI was small—only 7 out of 830. In 17 other cases, it was not possible to estimate the CCI SE because the bootstrap did not have enough clusters with data to run properly. We could not estimate absolute income for three countries because GDP or Gini index values were not available for the same year the survey had been carried out. Nevertheless, these are small numbers compared with the total number of countries and deciles studied. The attribution of absolute income might have been done for deciles instead of quintiles. We chose to use quintiles because there is some concern that the wealth index might not provide a completely comparable ranking of households to the ranking according to income.^16^ By using quintiles, we minimise errors in the attribution of income; and, as our results show, quintiles are perfectly adequate for comparing how countries fare in providing health coverage to different levels of income in their population.

In summary, we presented a comprehensive analysis of wealth-related health inequalities using innovative methods. In addition to confirming the magnitude of socioeconomic inequalities in RMNCH coverage, we provide compelling evidence that such inequalities are particularly driven by failure to reach the poorest women and children and identify a number of countries which—in spite of widespread poverty—are succeeding in leaving no one behind.

10.1136/bmjgh-2019-002229.supp2 Supplementary data
