# Supplementary material for: Are the poorest poor being left behind? Estimating global inequalities in reproductive, maternal, newborn and child health
Source: BMJ Glob Health. 2020 Jan 26;5(1):e002229. doi: 10.1136/bmjgh-2019-002229 (PMC7042578; doi:10.1136/bmjgh-2019-002229)
Supplement: Supplementary data [file bmjgh-2019-002229supp001.pdf]

Supplementary methods

The composite coverage index (CCI)

Our main outcome was the composite coverage index (CCI), a combined coverage indicator that considers the four stages of the reproductive, maternal, neonatal and child health (RMNCH) continuum of care. [3,4] The CCI is calculated as a weighted average of eight health interventions giving equal weight to each stage. The expression used for the calculation is

$$CCI = {}^{1}/_{4}\left(DFPSm + \frac{ANC4+SBA}{2} + \frac{BCG+2DPT3+MCV}{4} + \frac{ORS+CPNM}{2}\right)$$

where

1. DFPSm = demand for family planning satisfied with modern methods;
2. ANC4 = four or more antenatal care visits with any provider;
3. SBA = skilled birth attendant;
4. BCG = one dose of bacillus Calmette-Guérin vaccine;
5. DPT3 = three or more doses of diphtheria, tetanus and pertussis vaccine (or any polyvalent vaccine containing DPT);
6. MCV = at least one dose of a measles-containing-vaccine;
7. ORS = oral rehydration salts for diarrhoea;
8. CPNM = care-seeking for suspected pneumonia.

The standard error for the CCI was estimated through the bootstrapping resampling technique. A total of 50 samples with replacement were drawn from the whole sample and for each quantile and the standard error is based on the distribution of these samples' average.

The full definition of each indicator used in the CCI calculation, including numerator and denominator, is presented in the Table S1.

Table S1. Detailed definitions of indicators that compose the composite coverage index (CCI).

Indicator	Denominator	Numerator
DFPSm	Women aged 15-49 years either married or in a union who are in need for contraception	Women who are using (or whose partner is using) a modern contraceptive method
ANC4	Women 15-49 years who had a live birth in the previous 2 (MICS) or 3 (DHS) years	Women attending at least four antenatal care visits with any provider
SBA	All live births in the previous 2 (MICS) or 3 (DHS) years	Births delivered by a skilled health professional (doctor, nurse, midwife, or other country specific cadre)
BCG	Children alive aged 12-23/18-29/15-26 months (depending on the country specific immunization calendar)	Children who received the BCG vaccine
DPT3	Children alive aged 12-23/18-29/15-26 months (depending on the country specific immunization calendar)	Children who received at least 3 doses of DPT vaccine
MSL	Children alive aged 12-23/18-29/15-26 months (depending on the country specific immunization calendar)	Children who received at least one dose of measles vaccine
ORS	Children alive aged 0-59 months who presented with diarrhoea in the previous 2 weeks	Children with diarrhoea who received oral rehydration salts
CPNM	Children alive aged 0-59 months who presented symptoms of pneumonia in the previous 2 weeks	Children with suspected pneumonia who were taken to a health service from an appropriate health institution or provider

Wealth index and absolute income

The wealth classification used in the analysis is based on an asset index created through principal components analysis and adjusted for urban and rural area of residence. The variables used to calculate the score include household assets (e.g. cookstove, bicycle, car), building materials of the house (e.g. wood floor, brick walls, corrugated roof) and access to utilities (e.g. sanitation, electricity). The continuous score is provided with the original survey datasets and calculated according to a standard methodology. [5,6] It was then split into five equally sized groups (quintiles) and into ten equally sized groups (deciles) at the household level.

The attribution of absolute income values to households was done according to the methods described in Fink et al.[7] In summary, an income distribution for each country is generated by using the consumption share in the country's GDP and the Gini coefficient to generate the parameters for a log-normal distribution. [8] This distribution is then used to simulate the income distribution of a large number of observations, which, in turn, are divided into quintiles, deciles or centiles. The average calculated for each quantile from this simulation exercise is then used as the income value for the households, in constant 2011 international dollars adjusted by purchasing power parity (PPP).

Expected coverage for a given income level

The estimation of the average CCI coverage for a given level of absolute income in international dollars was done through a linear multilevel model where the outcome was the CCI and the predictor was the log-transformed income. Quintiles were level 1 units and countries level 2 units in the multilevel model. The adjusted CCI coverage and its standard error was then estimated from the resulting model.

Inequality measures

Absolute inequality across the wealth distribution was measured using the slope index of inequality (SII). The SII was estimated through individual-level logistic regression with the intervention coverage (yes or no) as the outcome and the wealth quintiles as the predictor. The difference between the extremes of the wealth distribution is then estimated using the resulting model. When used for coverage indicators, the SII can vary from -100 to +100, where zero means absence of inequality. A positive value indicates that the richer groups present higher coverage than the poorer groups, while negative values mean the opposite.[8]

Statistics package

All analyses were carried out with Stata (StataCorp. 2017. Stata Statistical Software: Release 15. College Station, TX: StataCorp LLC). The individual level analyses (such as estimation of the CCI and components) accounted for the survey design by adjusting for the clustered nature of the sample, stratification and sample weights. Country-level (or quintile-level) analyses were not weighted by population size so that each country (or quintile) has the same weight in the analyses (e.g. correlation estimates).

Supplementary results

Supplementary results are presented in a supplementary Excel spreadsheet containing Table S1, Table S2, Table S3 and Figure S1.

References

1 USAID (United States Agency for International Development). Demographic and Health Surveys (DHS). [Internet]. www.dhsprogram.com (accessed Jun 2019)

2 UNICEF (United Nations Children's Fund). Multiple Indicator Cluster Surveys (MICS). [Internet]. mics.unicef.org (accessed Jun 2019)

3 Restrepo-Mendez MC, Barros AJ, Requejo J, *et al.* Progress in reducing inequalities in reproductive, maternal, newborn,' and child health in Latin America and the Caribbean: an unfinished agenda. *Rev Panam Salud Publica* 2015;**38**:9–16.internal-pdf://197.133.93.151/Restrepo-Mendez-2015.pdf

4 Barros AJD, Victora CG. Measuring coverage in MNCH: determining and interpreting inequalities in coverage of maternal, newborn, and child health interventions. *PLoS Med* 2013;**10**:e1001390. doi:10.1371/journal.pmed.1001390

5 Rutstein SO. *The DHS Wealth Index: Approaches for rural and urban areas*. Calverton, Maryland: ORC Macro 2008.

6 Rutstein SO, Johnson K. *The DHS wealth index: comparative reports n° 6*. Calverton, Maryland: ORC Macro 2004.

7 Fink G, Victora CG, Harttgen K, *et al.* Predicted Absolute Incomes Rather Than Wealth Quintiles : A Comparative Assessment Using Child Stunting Data From National Surveys. *Am J Public Health* 2017;:1–6. doi:10.2105/AJPH.2017.303657

8 Harttgen K, Vollmer S. Using an asset index to simulate household income. *Econ Lett* 2013;**121**:257–62. doi:10.1016/J.ECONLET.2013.08.014
